# Relapsing–Remitting Multiple Sclerosis Is Characterized by a T Follicular Cell Pro-Inflammatory Shift, Reverted by Dimethyl Fumarate Treatment

**DOI:** 10.3389/fimmu.2018.01097

**Published:** 2018-05-29

**Authors:** Vanesa Cunill, Margarita Massot, Antonio Clemente, Carmen Calles, Valero Andreu, Vanessa Núñez, Antonio López-Gómez, Rosa María Díaz, María de los Reyes Jiménez, Jaime Pons, Cristòfol Vives-Bauzà, Joana Maria Ferrer

**Affiliations:** ^1^Immunology Department, Hospital Universitari Son Espases, Palma, Spain; ^2^Human Immunopathology Research Laboratory, Institut d’Investigació Sanitària de les Illes Balears (IdISBa), Palma, Spain; ^3^Neurology Department, Hospital Universitari Son Espases, Palma, Spain; ^4^Clinical Trials and Methodology Support Platform, Institut d’Investigació Sanitària de les Illes Balears (IdISBa), Palma, Spain; ^5^Research Unit, Institut d’Investigació Sanitària de les Illes Balears and Hospital Universitari Son Espases, Palma, Spain

**Keywords:** multiple sclerosis, dimethyl fumarate, follicular T cells, cTfh17.1, B cells

## Abstract

Multiple sclerosis (MS) is considered a T cell-mediated autoimmune disease, although several evidences also demonstrate a B cell involvement in its etiology. Follicular T helper (Tfh) cells, a CXCR5-expressing CD4+ T cell subpopulation, are essential in the regulation of B cell differentiation and maintenance of humoral immunity. Alterations in circulating (c)Tfh distribution and/or function have been associated with autoimmune diseases including MS. Dimethyl fumarate (DMF) is a recently approved first-line treatment for relapsing–remitting MS (RRMS) patients whose mechanism of action is not completely understood. The aim of our study was to compare cTfh subpopulations between RRMS patients and healthy subjects and evaluate the impact of DMF treatment on these subpopulations, relating them to changes in B cells and humoral response. We analyzed, by flow cytometry, the distribution of cTfh1 (CXCR3+CCR6−), cTfh2 (CXCR3−CCR6−), cTfh17 (CXCR3−CCR6+), and the recently described cTfh17.1 (CXCR3+CCR6+) subpopulations of CD4+ Tfh (CD45RA−CXCR5+) cells in a cohort of 29 untreated RRMS compared to healthy subjects. CD4+ non-follicular T helper (Th) cells (CD45RA−CXCR5−) were also studied. We also evaluated the effect of DMF treatment on these subpopulations after 6 and 12 months treatment. Untreated RRMS patients presented higher percentages of cTfh17.1 cells and lower percentages of cTfh2 cells consistent with a pro-inflammatory bias compared to healthy subjects. DMF treatment induced a progressive increase in cTfh2 cells, accompanied by a decrease in cTfh1 and the pathogenic cTfh17.1 cells. A similar decrease of non-follicular Th1 and Th17.1 cells in addition to an increase in the anti-inflammatory Th2 subpopulation were also detected upon DMF treatment, accompanied by an increase in naïve B cells and a decrease in switched memory B cells and serum levels of IgA, IgG2, and IgG3. Interestingly, this effect was not observed in three patients in whom DMF had to be discontinued due to an absence of clinical response. Our results demonstrate a possibly pathogenic cTfh pro-inflammatory profile in RRMS patients, defined by high cTfh17.1 and low cTfh2 subpopulations that is reverted by DMF treatment. Monitoring cTfh subsets during treatment may become a biological marker of DMF effectiveness.

## Introduction

Multiple sclerosis (MS), one of the most common causes of neurological disability in young adults, is a chronic progressive neurodegenerative autoimmune disease of the central nervous system (CNS), which leads to inflammation, demyelination, and axonal damage in the brain and spinal cord (1). Based on symptoms onset and clinical course, two main types of MS can be described: relapsing–remitting MS (RRMS) and progressive MS. RRMS affects 85% MS patients and is characterized by young adulthood onset episodes of acute exacerbations followed by complete or partial recovery. Many of these patients develop a secondary progressive form of MS (PSMS) with gradual progression of disability. The annual conversion rate into PSMS is 2.5%, approximately. Primarily progressive MS, characterized by continuous worsening without relapses, accounts for only 15% of MS patients (2).

Multiple sclerosis etiology is still incompletely understood. Both CD4+ and CD8+ T cells and B cells have been described as important players in MS pathogenesis. Historically, autoreactive IFNγ-producing T helper (Th) 1 cells were considered the main mediators of inflammation causing MS lesions (3). This model was challenged with the discovery of interleukin (IL)-17-producing Th17 cells. Th17 cells secrete pro-inflammatory cytokines (IL-17 and IL-6) and express CCR6, the CCL20 ligand expressed on vascular endothelial cells that allows them to pass through the blood–brain barrier (4). However, although MS is thought to be a T cell-mediated disease, several lines of evidence demonstrate the involvement of B cells in disease course. The presence of oligoclonal bands in cerebrospinal fluid in as many as 95% diagnosed patients and the presence of B cells, plasma cells, and meningeal B-cell follicles in the CNS, point to the involvement of B cells and antibody production in MS (5). Moreover, clinical trials using B cell-depleting therapies suggests that a decrease in B cell antigen presenting ability and a change in B cells cytokine production contribute to reduce MS activity (6).

Significant advances in the development of disease-modifying drugs for RRMS have been achieved. Dimethyl fumarate (DMF) is an oral fumaric acid ester approved by the FDA and EMA in 2013 as a first-line treatment for RRMS. Clinical trials of DMF-treated RRMS patients showed significant reductions in clinical relapses and MRI evidence of inflammatory disease (7). The mechanism of action of DMF is not completely understood, but it is known that DMF reduces oxidative stress and modulates nuclear factor κappa B, which could have anti-inflammatory effects, mainly mediated through the activation of the Nrf2 pathway (8). Recent studies have shown a reduction of CD4+ T, CD8+ T, B, and NKT cells, and a shift in Th subpopulations in RRMS patients treated with DMF (9).

The follicular T helper (Tfh) cells are a CD4+T cell subpopulation essential in the regulation of humoral immunity, specialized in supporting B cell maturation and immunoglobulin production in secondary lymphoid organs. Tfh cells were first described as a CXCR5-expressing population localizing in “tonsillar” follicles (10, 11). The discovery of a circulating counterpart of this population has allowed investigating their relevance in health and disease (12). Morita et al. originally demonstrated that analogous to non-follicular Th cells, circulating Tfh (cTfh) cells can be classified, according to the expression of CXCR3 and CCR6, into cTfh1 (CXCR3+CCR6−), cTfh2 (CXCR3−CCR6−), and cTfh17 (CXCR3−CCR6+) whose differentiation relies on distinct transcription factors (12). These subpopulations produce a different set of cytokines and exert different B helper cell capabilities. Alterations in cTfh function and/or distribution have been associated with the pathogenesis of many autoimmune diseases, including MS (13, 14) and also with infectious diseases and monogenic immunodeficiencies (15). A CXCR3+CCR6+ Th subpopulation that rivals with Th1 in IFNγ production, but also produces IL-17, has been described as Th17.1 and identified in sarcoidosis and Crohn’s disease (16–18). A follicular CXCR5-expressing counterpart (cTfh17.1) has also been recently identified in common variable immunodeficiency (19).

The aim of the present study was to compare cTfh subpopulations between RRMS patients and healthy controls, to evaluate the impact of DMF treatment on these subpopulations and its relationship with changes in B cell distribution and clinical evolution.

## Materials and Methods

### Patients

29 patients, 19 females, and 10 males, diagnosed with RRMS defined by 2010 McDonald criteria (20) were included in the study. All RRMS patients were treated with 240 mg DMF twice a day and followed 6 and 12 months after starting treatment. 35 age- and sex-matched healthy subjects were included as controls.

Patients mean age was 41.2 years (range 26–60). Mean time since first MS appearing symptoms was 6.1 ± 5.1 years. Clinical relapses were evaluated in our cohort before DMF treatment. The number of clinical relapses 1 and 2 years previous to DMF treatment was (0.58 ± 0.5) and (0.79 ± 0.6), respectively. 15 patients had ≥1 gadolinium-enhancing (Gd+) lesions, 22 had >10 supratentorial T2*-weighted lesions, and 18 > 1 infratentorial T2*-weighted lesions in baseline MRI findings.

The mean Expanded Disability Status Scale (EDSS) was 1.8 ± 1.3 prior to DMF treatment, 1.8 ± 1.2 after 6 months treatment, and 2.1 ± 1.3 after 12 months treatment, the change not proving significant (paired test; *p* = 0.82). 10 patients diagnosed with RRMS had not received any previous treatment and 20 had received first line drugs (14 interferon-β and 5 glatiramer-acetate) before starting DMF treatment.

The 14 patients included in the longitudinal cohort displayed similar demographic and clinical characteristics.

The study was conducted according to the ethical guidelines of the 1975 Declaration of Helsinki and approved by CEIC (Balearic Islands Clinical Research Ethics Committee). Written informed consent was obtained from all subjects.

### Flow Cytometry

Cell surface markers expression was analyzed by flow cytometry using a Navios cytometer and data evaluated using Kaluza software (Beckman Coulter, USA).

A surface staining protocol was performed to analyze membrane antigen expression in lymphocytes subpopulations. Briefly, 100 µL of peripheral fresh whole blood were incubated 20 min at room temperature (25°C) with different fluorochrome-conjugated monoclonal antibodies combinations. Red blood cells were lysed, white cells fixed using TQ-Prep System (Beckman Coulter), and 100 µl of fluorospheres Flow-Count™ (Fluorospheres, Beckman Coulter) were added to calculate cell concentration, previous to flow cytometric analysis.

Combinations of the following antibodies were used: anti-CD45-FITC, anti-CD4-PE, anti-CD8-ECD, anti-CD3-PCy5, anti-CD56-PCy5, anti-CD3-PCy7, and anti-CD45RA-ECD (all from Coulter Immunotech, France) to evaluate T and NK cells; anti-CD19-ECD, anti-CD27-PCy7, anti-CD21-FITC, anti-CD24-PCy5, anti-CD38-PCy7 (all from Coulter Immunotech, France) and anti-IgD-FITC (Dako, Spain) to study B cells; anti-CD4-PCy5, anti-CD45RA-ECD (from Coulter Immunotech, France), anti-CXCR5-PE (R&D Systems, Spain), anti-CXCR3-FITC and anti-CCR6-PCy7 (Biolegend, USA) to evaluate Th and cTfh subpopulations.

### Nephelometry

Serum total IgG, IgA, IgM levels quantification and IgG subclass profile (IgG1, IgG2, IgG3, IgG4) were assayed by nephelometry in a BN II nephelometer (Siemens, Germany). Commercially available kits (Siemens, Germany) were used according to manufacturer’s instructions protocol. The intra and inter-assay coefficients of variations were less than 5%.

### Statistical Analysis

Statistical analysis was performed using GraphPad Prism version 4.0 software. Data are expressed as mean values. The Mann–Whitney *U*-test was used to compare differences between controls and untreated RRMS patients or controls and RRMS 12 months DMF-treated patients. The Kruskal–Wallis test was used to compare differences between subgroups of RRMS patients.

For longitudinal samples, Wilcoxon and Friedman test were used to compare paired data. We used Wilcoxon test to compare differences between untreated and 12 months DMF-treated RRMS patients. Friedman test along with a Dunn multiple comparison test were applied to assess differences between untreated RRMS patients and 6 and 12 months DMF-treated RRMS patients. A *p*-value ≤ 0.05 was considered statistically significant.

## Results

### Peripheral Blood Lymphocyte Populations in Untreated RRMS Patients

Percentages and absolute counts of CD4+ and CD8+ T cells, B, and NK cells in untreated RRMS patients were within reported ranges for Caucasians (21, 22) (Table 1).

**Table 1:** Lymphocytes subpopulations of untreated multiple sclerosis (MS) patients.

Patients	CD3+%	CD4+%	CD4+cells/μL	CD8+%	CD8+cells/μL	CD19+%	CD19+cells/μL	CD3−CD56+%	CD3−CD56+cells/μL	CD19+ (%)					
										CD21^low^	IgD+CD27−	IgD+CD27+	IgD−CD27+	CD38^high^CD24^high^	CD38^high^CD24−
1	66	45	908	21	433	16	323	16	323	10	48	32	17	1.3	0.3
2	71	50	641	19	250	15	197	10	132	4	75	6	9	3.39	0.1
3	66	50	1,105	14	318	23	497	9	199	1.3	74	9	9	12	0
4	87	57	738	28	358	5	69	7	94	NA	NA	NA	NA	NA	NA
5	77	45	803	31	378	11	197	11	197	6	67	5	15	NA	NA
6	74	56	820	16	237	14	210	12	170	3	60	5	27	2.85	0
7	78	62	953	14	218	11	162	8	126	9	26	8	50	1.03	0.5
8	83	45	742	36	729	11	182	4	72	8	43	13	33	3.89	0
9	73	49	1,353	22	578	16	439	11	315	19	51	13	24	2.2	0.1
10	72	45	892	22	458	16	313	10	193	7	61	9	19	5.53	0
11	65	42	729	20	338	5	94	26	454	10	59	6	25	3.3	0
12	76	51	509	22	209	15	152	8	77	7	59	6	18	6.89	0.2
13	74	45	485	27	269	16	169	10	102	8	53	4	31	8.7	0
14	68	51	NA	15	NA	21	NA	NA	NA	NA	NA	NA	NA	2.17	1.29
15	76	61	1,136	13	241	13	233	8	140	10	28	6	59	0.7	2.6
16	81	57	1,333	20	558	9	202	7	156	NA	65	8	20	5.72	0
17	66	47	677	18	257	6	88	25	354	19	46	17	27	2.93	0.37
18	73	49	768	19	329	14	219	13	203	7	54	7	27	NA	NA
19	63	46	503	17	181	27	298	8	84	11	72	4	7	12.3	0.1
20	72	55	1,073	17	314	18	348	8	152	4	62	8	19	4	0
21	72	37	533	34	482	16	222	8	109	6	70	10	14	6.27	0.4
22	80	54	924	24	396	9	158	7	114	11	22	8	55	1.61	0.06
23	82	50	1,485	27	844	10	307	7	209	9	58	13	18	5.58	0.15
24	82	52	359	27	182	9	63	6	40	15	28	11	41	2.24	1
25	80	57	1,249	20	417	9	202	8	177	10	42	17	34	4.72	0.44
26	70	43	NA	21	NA	12	NA	10	NA	10	45	13	33	7.32	0.3
27	79	63	NA	14	NA	9	NA	9	NA	6	54	13	19	3.08	0.43
28	77	47	998	23	486	9	191	17	362	NA	NA	NA	NA	7.2	0
29	87	69	1,091	16	263	10	159	3	47	NA	NA	NA	NA	11.7	0.2
Reference ranges	53–83	30–59	490–1,640	10–40	170–880	5–21	80–490	5–32	80–690	1–8	53–86	3–13	4–22	0.9–6.3	0.1–1.5

*T (CD3+, CD3+CD4+ and CD3+CD8+), NK (CD3−CD56+), and B (CD19+) cells were evaluated as percentages from total lymphocytes and as absolute counts (cells/μL). Percentages of CD21^low^, naïve IgD+CD27−, non-switched memory IgD+CD27+, switched memory IgD−CD27+, transitional CD38^high^CD24^high^ and plasmablasts CD38^high^CD24− B cell subpopulations (referred to total CD19+ B lymphocytes) of MS patients. NA, not available*.

**Figure 1:**
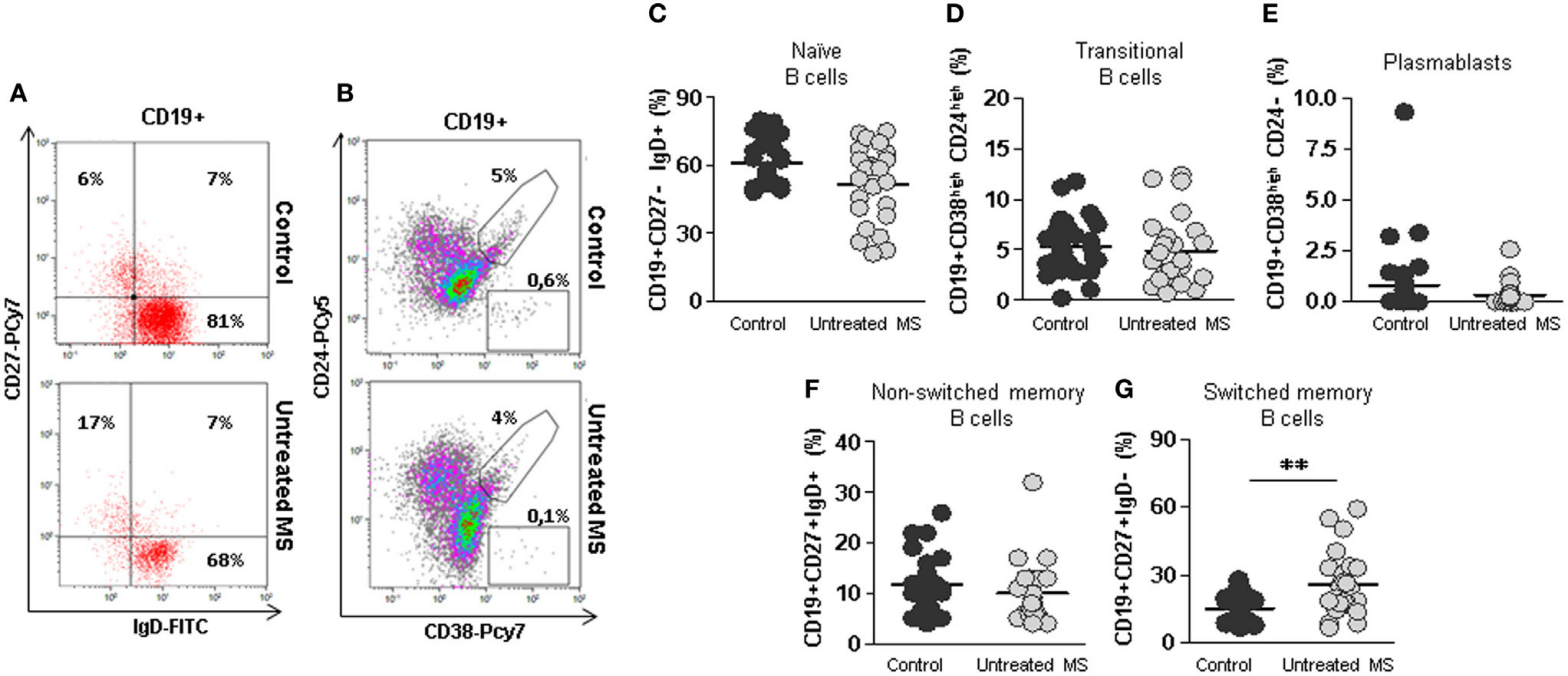
Subpopulations of B cells from untreated multiple sclerosis (MS) patients. **(A)** Dot plots of the percentages of peripheral naïve CD19+CD27−IgD+ (lower right quadrant), non-switched memory CD19+CD27+IgD+ (upper right quadrant), and switched memory CD19+CD27+IgD− (upper left quadrant) B cells on total CD19+ gated B cells from a representative untreated MS patient (lower row) and control (upper row). **(B)** Dot plots of the percentages of peripheral transitional CD19+CD38^high^CD24^high^ B cells on total CD19+ gated B cells from a representative untreated MS patient (lower row) and control (upper row). **(C–G)** Percentages of naïve CD19+CD27−IgD+, transitional CD19+CD38^high^CD24^high^, plasmablasts CD19+CD38^high^CD24−, non-switched memory CD19+CD27+IgD+, and switched memory CD19+CD27+IgD− B cells from controls (dark gray circles) and untreated MS patients (light gray circles). Mann–Whitney *U*-test *p*-values: **p < 0.01.

However, B cells subsets distribution was altered (Figures 1A,B). When we compared B cells subsets from untreated RRMS patients with those of our healthy subjects’ cohort, we observed a trend toward decreased percentages of naïve B cells (CD19+CD27−IgD+) in untreated RRMS patients (52 vs. 60.7%; *p* = 0.09) (Figure 1C). The percentages of transitional (CD19+CD38^high^CD24^high^), plasmablasts (CD19+CD38^high^CD24−) and non-switched memory (CD19+CD27+IgD+) B cells were similar between untreated RRMS patients and controls (Figures 1C–F). However, we found a significantly higher percentage of switched-memory (CD19+CD27+IgD−CD27+) B cells in untreated RRMS patients (25.9 vs. 16.5%; *p* < 0.01) compared to healthy subjects (Figure 1G).

### Selectively Increased cTfh17.1 and Decreased cTfh2 Effector Cells in Untreated RRMS Patients

**Figure 2:**
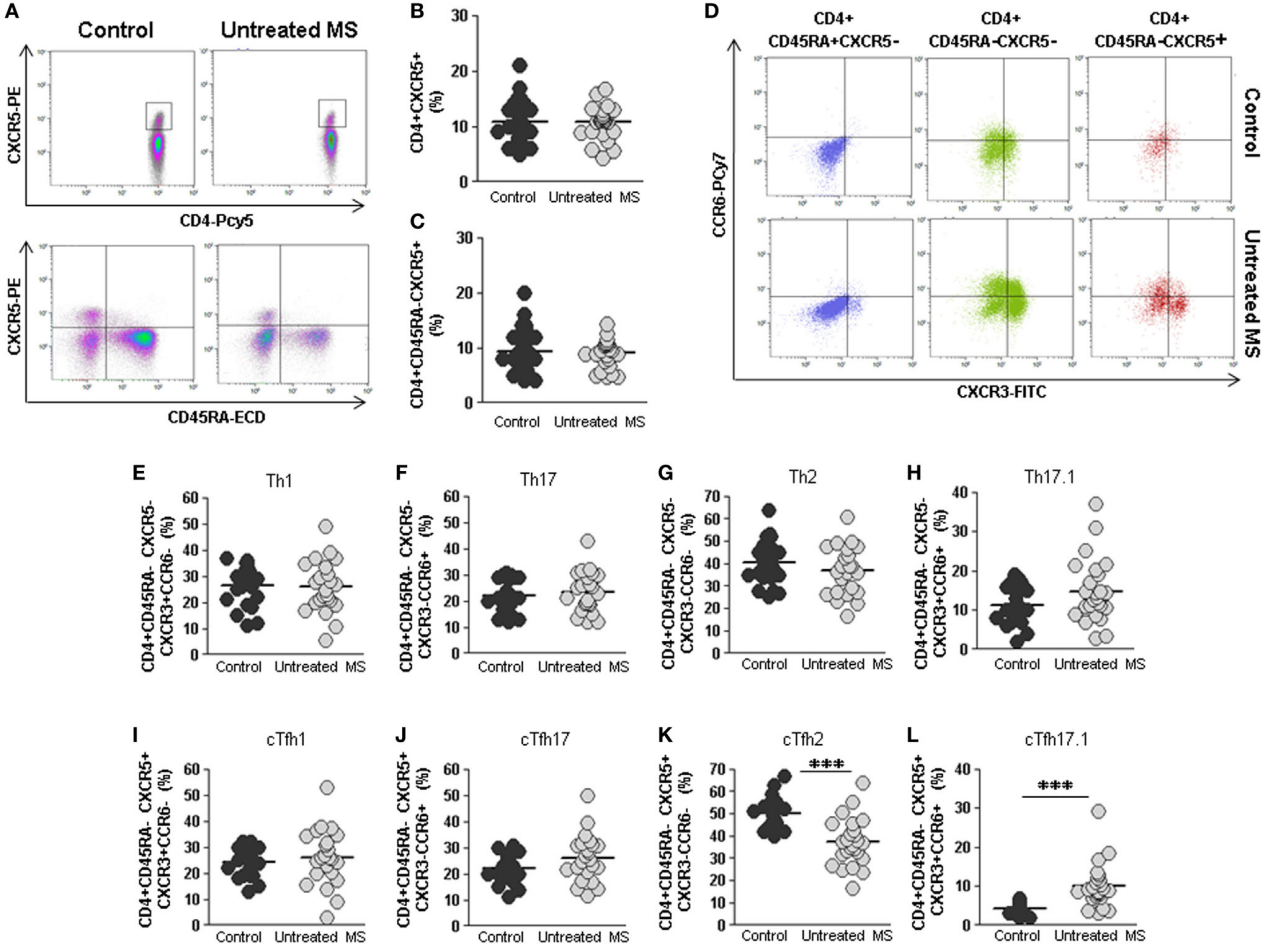
Total cTfh cells and subpopulations of Th and cTfh cells from untreated multiple sclerosis (MS) patients. **(A)** Density plots of the percentage of total follicular CD4+CXCR5+ (upper plots) and memory follicular CD4+CD45RA−CXCR5+ (lower plots) cells from a representative untreated MS patient (right) and control (left). **(B)** Percentages of total follicular CD4+CXCR5+ cells from controls (dark gray circles) and untreated MS patients (light gray circles). **(C)** Percentages of total memory follicular CD4+ CD45RA−CXCR5+ cells from healthy controls (dark gray circles) and untreated MS patients (light gray circles). **(D)** Dot-plots of the percentage of Th1 CXCR3+CCR6− (lower right quadrant), Th17 CXCR3−CCR6+ (upper left quadrant), Th2 CXCR3−CCR6− (lower left quadrant), and Th17.1 CXCR3+CCR6+ (upper right quadrant) subpopulations on circulating naïve CD4+CD45RA+CXCR5− (left), non-follicular CD4+CD45RA−CXCR5− (middle), and follicular CD4+CD45RA−CXCR5+ (right) cells from a representative untreated MS patient (lower row) and control (upper row). **(E–H)** Percentages of Th1, Th17, Th2, and Th17.1 non-follicular T cells from controls (dark gray circles) and untreated MS patients (light gray circles). **(I–L)** Percentages of cTfh1, cTfh17, cTfh2, and cTfh17.1 follicular T cells from controls (dark gray circles) and untreated MS patients (light gray circles). Mann–Whitney *U*-test *p*-values: ***p < 0.001.

No differences were found in the percentage of total CD4+CXCR5+ cTfh cells between untreated RRMS patients and controls (10.8 vs. 10.8%) (Figures 2A,B). Several studies have shown that cTfh cells are contained within the memory CD45RA−CD4+ T cells. No differences were found in the percentage of circulating cTfh cells, analyzed as memory CD4+CD45RA−CXCR5+, in untreated RRMS patients compared to controls (9.1 vs. 9.3%) (Figure 2C).

We further studied the distribution of different subsets of circulating effector T helper non-follicular (Th) CD4+CD45RA−CXCR5− and follicular (cTfh) CD4+CD45RA−CXCR5+ cells, identified according to the expression of CXCR3 and CCR6 markers in untreated RRMS patients and controls: Th1/cTfh1 (CXCR3+CCR6−), Th2/cTfh2 (CXCR3−CCR6−), Th17/cTfh17 (CXCR3−CCR6+), and Th17.1/cTfh17.1 (CXCR3+CCR6+) (Figure 2D).

No differences were found when Th1, Th17, and Th2 non-follicular subpopulations were compared (Figures 2E–G). There was a trend toward higher percentage of Th17.1 cells in untreated RRMS patients, although it was not statistically significant (Figure 2H). Interestingly, when cTfh1, cTfh17, and cTfh2 CD4+CD45RA−CXCR5+ subpopulations were evaluated, we found a lower percentage of cTfh2 cells in untreated RRMS patients compared to controls (37.4 vs. 49.9%; *p* < 0.001) (Figure 2K), whereas the percentage of cTfh17.1 was significantly increased (10.0 vs. 4.2%; *p* < 0.001) (Figure 2L). No differences were found in cTfh1 and cTfh17 subpopulations (Figures 2I,J).

To rule out the possibility that differences found in switched-memory B cells or cTfh17.1 and cTfh2 subpopulations were caused by previous treatments, these subpopulations were compared between RRMS patients who had or not received previous disease-modifying treatments. Interestingly, no differences were found in the percentages of switched-memory B, cTh2 and cTh17.1 cells (data not shown).

These results demonstrate a clear selective pro-inflammatory shift in cTfh subpopulations and B cells in RRMS patients, not present in the non-follicular Th subpopulations, which is not caused by previous treatments.

### DMF Treatment Reduces CD4+ and CD8+ T, NK, and B Cells Counts

**Figure 3:**
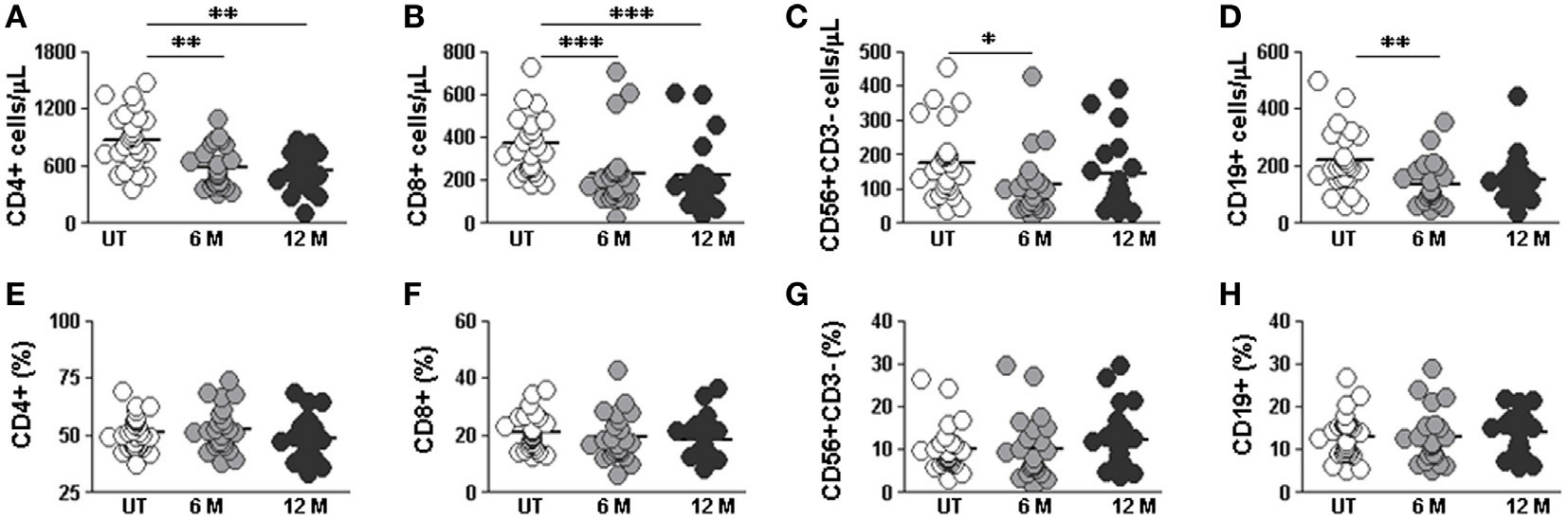
Effect of dimethyl fumarate on CD4, CD8, NK, and B cells from multiple sclerosis (MS) patients. **(A–D)** Cell counts and **(E–H)** percentages of peripheral CD3+CD4+, CD3+CD8+, CD56+CD3− (NK), and CD19+ **(B)** lymphocytes from untreated (UT: white circles), 6 months treated (6 months: light gray circles), and 12 months treated (12 months: dark gray circles) MS patients. Kruskal–Wallis test *p*-values: *p < 0.05, **p < 0.01, and ***p < 0.001.

We next studied the effect of DMF treatment on the different lymphocyte subpopulations. In order to do so, we compared lymphocyte subpopulations in “short term” 6 months and “long term” 12 months treated RRMS patients with the ones of untreated RRMS patients. We found lower absolute numbers of CD4+ T cells both after 6 and 12 months treatment compared to untreated patients (588.4 vs. 877.2 cells/μL; *p* < 0.01) and (555.8 vs. 877.2 cells/μL; *p* < 0.01), respectively (Figure 3A). CD8+ T cells counts were lower in 6 months (230.0 vs. 374.0 cells/μL; *p* < 0.001) and 12 months treated (223.3 vs. 374.0 cells/μL; *p* < 0.001) (Figure 3B) compared to untreated patients. NK and B cells counts were also lower in 6 months treated compared to untreated patients (111.6 vs.177.0 cells/μL and 137.7 vs. 219.0 cells/μL; *p* < 0.05 and *p* < 0.01, respectively) (Figures 3C,D). This is consistent with the reported lymphopenia described in DMF-treated RRMS patients (9). In agreement with previous studies (9), we did not find differences in the percentage of any lymphocyte subset between 6 and 12 months treated compared to untreated patients (Figures 3E–H).

### DMF Treatment Alters B Cells Distribution and Immunoglobulins Levels

**Figure 4:**
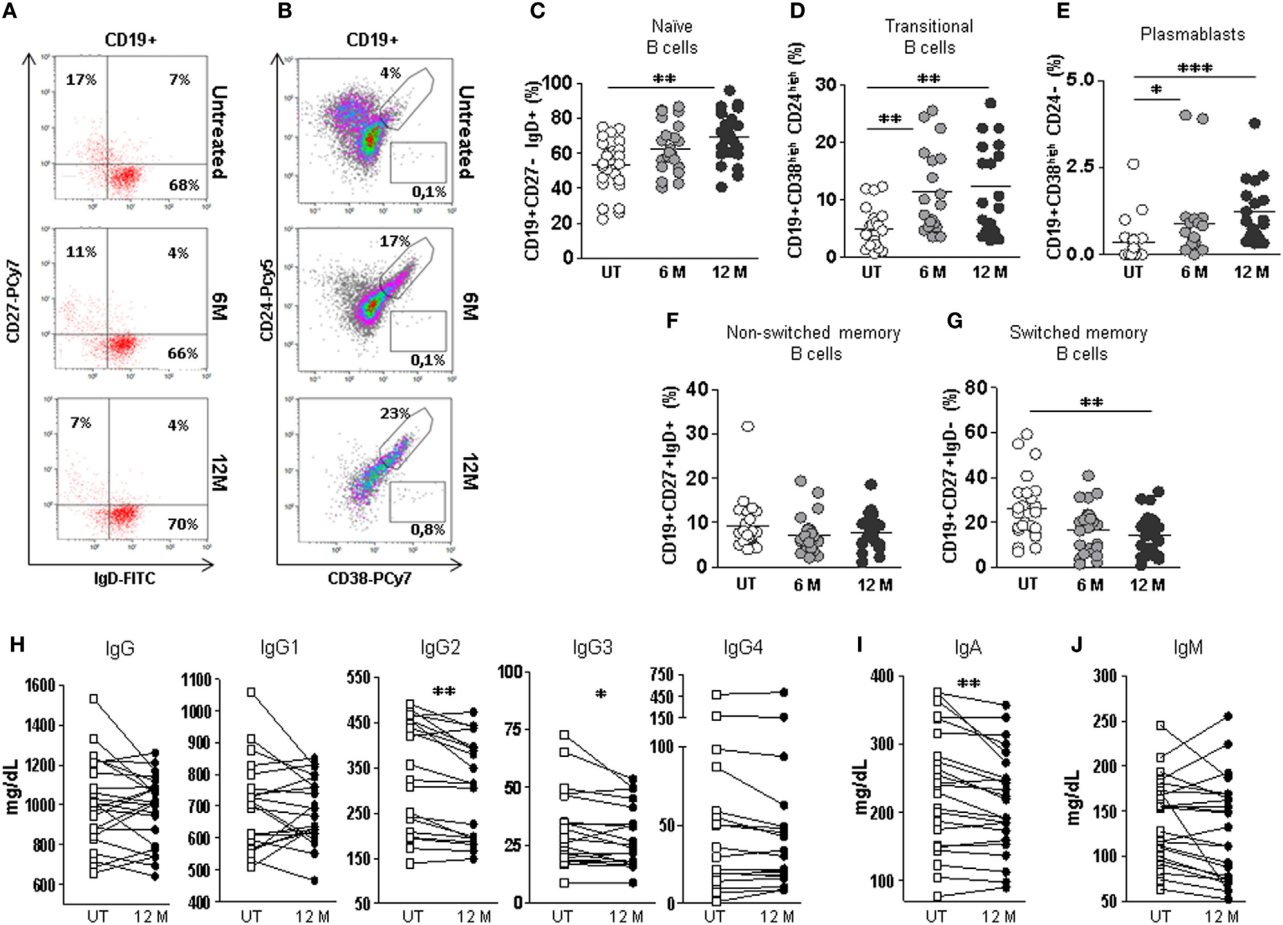
Effect of dimethyl fumarate on B cells subpopulations and serum levels of immunoglobulins in multiple sclerosis (MS) patients. **(A)** Dot-plots of the percentages of peripheral naïve CD19+CD27−IgD+ (lower right quadrant), non-switched memory CD19+CD27+IgD+ (upper right quadrant), and switched memory CD19+CD27+IgD− (upper left quadrant) B cells on total CD19+ gated B cells from a representative untreated (upper row), 6 months treated (6 M: middle row), and 12 months treated (12 M: lower row) MS patient. **(B)** Dot plots of the percentages of peripheral transitional CD19+CD38^high^CD24^high^ B cells on total CD19+ gated B cells from a representative untreated (upper row), 6 months treated (6 M: middle row), and 12 months treated (12 M: lower row) MS patient. **(C–G)** Percentages of peripheral naïve CD19+CD27−IgD+, transitional CD19+CD38^high^CD24^high^, plasmablasts CD19+CD38^high^CD24−, non-switched memory CD19+CD27+IgD+, and switched memory CD19+CD27+IgD− B cells from untreated (UT: white circles), 6 months treated (6 M: light gray circles), and 12 months treated (12 M: dark gray circles) MS patients. Kruskal–Wallis test *p*-values: *p < 0.05, **p < 0.01, and ***p < 0.001. **(H–J)** Serum IgG, IgG1, IgG2, IgG3, IgG4, IgA, and IgM levels (mg/dL) from MS patients before treatment (UT: white squares) and 12 months (12 M: dark gray circles) after treatment. Successive repeated measures on the same individual are represented by connecting lines. Wilcoxon paired test: *p*-values: *p < 0.05, **p < 0.01.

The distribution of B cells subpopulations was significantly altered by DMF treatment (Figures 4A,B). When DMF-treated RRMS patients were compared with untreated patients, we found significantly increased percentages of naïve B cells after 12 months treatment (69.5 vs. 52.9%; *p* < 0.01), transitional B cells after 6 and 12 months treatment (11.4 vs. 4.8% and 12.3 vs. 4.8%; *p* < 0.01, respectively) and plasmablasts after 6 and 12 months treatment (0.8 vs. 0.3% and 1.2 vs. 0.3%; *p* < 0.05 and *p* < 0.001, respectively) (Figures 4C–E). On the contrary, a significant decrease in switched memory B cells was found in 12 months treated compared to untreated RRMS patients (14.3 vs. 25.9%; *p* < 0.01) (Figure 4G). No differences were found in non-switched memory B cells (Figure 4F).

To evaluate if these B cell subpopulation changes had any observable humoral consequence, we quantified immunoglobulin levels in serum samples from 23 longitudinally followed patients before and after 12 months DMF treatment. We found significantly lower levels of IgA in 12 months treated patients compared to untreated patients (Wilcoxon paired test; *p* < 0.01) (Figure 4I). No differences were found when the effect of DMF on IgG and IgM serum levels was evaluated (Figures 4H,J).

We also studied if DMF induced changes in the serum IgG subclass profile (IgG1, IgG2, IgG3 and IgG4). Interestingly, although IgG levels were similar before and after 12 months DMF treatment, the IgG subclass profile was altered. We found significantly lower levels of IgG3 and IgG2 in the serum of 12 months DMF-treated RRMS patients compared to untreated patients (Wilcoxon paired test; *p* < 0.05 and *p* < 0.01, respectively) (Figure 4H).

### DMF Treatment Reduces Both Absolute Numbers and Percentage of cTfh Cells in RRMS Patients

**Figure 5:**
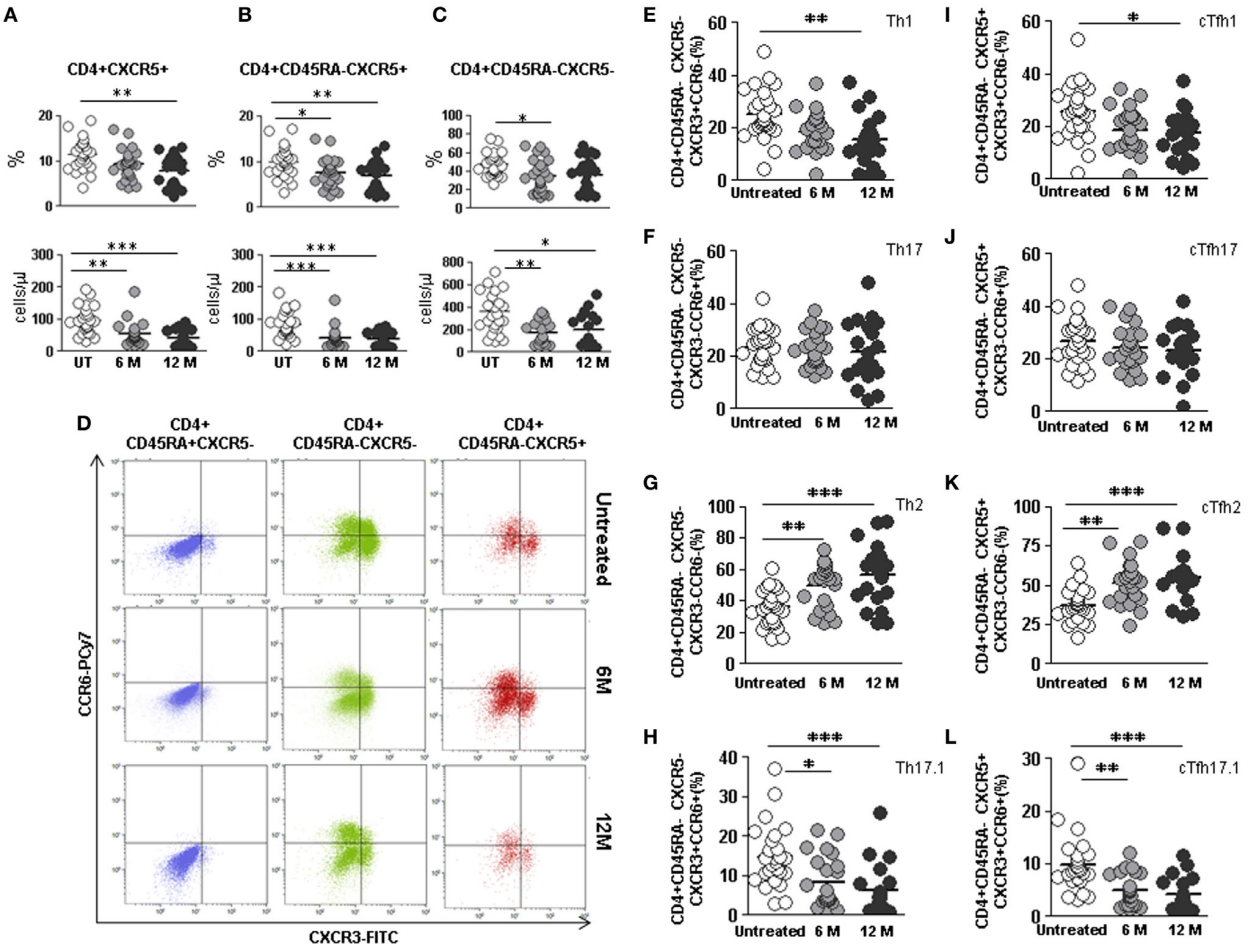
Effect of dimethyl fumarate on total cTfh cells and subpopulations of Th and cTfh cells from multiple sclerosis (MS) patients. **(A–C)** Total follicular CD4+CXCR5+, memory follicular CD4+CD45RA−CXCR5+, and memory non-follicular CD4+CD45RA−CXCR5− percentages (upper panels) and cell counts (lower panels) from untreated (white circles), 6 months treated (6 M: light gray circles), and 12 months treated (12 M: dark gray circles) MS patients. **(D)** Dot-plots of the percentage of Th1 CXCR3+CCR6− (lower right quadrant), Th17 CXCR3−CCR6+ (upper left quadrant), Th2 CXCR3−CCR6− (lower left quadrant), and Th17.1 CXCR3+CCR6+ (upper right quadrant) subpopulations on circulating naïve CD4+CD45RA+CXCR5− (left), non-follicular CD4+CD45RA−CXCR5− (middle), and follicular CD4+CD45RA−CXCR5+ (right) cells from a representative untreated (upper row), 6 months treated (6 M: middle row), and 12 months treated (12 M: lower row) MS patient. **(E–H)** Percentages of Th1, Th17, Th2, and Th17.1 non-follicular T cells and **(I–L)** cTfh1, cTfh17, cTfh2, and cTfh17.1 follicular T cells from untreated (white circles), 6 months treated (6 M: light gray circles), and 12 months treated (12 M: dark gray circles) MS patients. Kruskal–Wallis test *p*-values: *p < 0.05, **p < 0.01, and ***p < 0.001.

The percentage of CD4+CXCR5+ cTfh cells was lower in 12 months DMF-treated compared to untreated RRMS patients (7.7 vs. 11.4%; *p* < 0.01) (Figure 5A, upper panel). We also found lower cTfh absolute counts in 6 months treated (54.8 vs. 97.0 cells/μL; *p* < 0.01) and 12 months (41.8 vs. 97.0 cells/μL; *p* < 0.001) compared to untreated RRMS patients (Figure 5A, lower panel).

Lower memory CD4+CD45RA−CXCR5+ cTfh cells percentages and absolute counts were also found in 6 months (7.5 vs. 9.9%; *p* < 0.05 and 43.0 vs. 84.7 cells/μL, *p* < 0.001, respectively) and 12 months treated RRMS patients (6.9 vs. 9.9%; *p* < 0.01 and 37.2 vs. 84.7 cells/μL *p* < 0.001, respectively) once compared to untreated patients (Figure 5B).

The percentage of non-follicular CD4+CD45RA−CXCR5− Th cells only differed after 6 months DMF treatment (34.1 vs. 47.0%; *p* < 0.05) (Figure 5C, upper panel), although the absolute counts significantly decreased after 6 and 12 months DMF treatment (170.2 vs. 361.8 cells/μL and 196.0 vs. 361.8 cells/μL; *p* < 0.01 and *p* < 0.05, respectively) (Figure 5C, lower panel). No differences were found when naïve CD4+CD45RA+CXCR5− Th cells subpopulations were compared (data not shown).

### Increase of cTfh2 and Decrease of cTfh1 and cTfh17.1 Effector Cells in DMF-Treated RRMS Patients

We next compared the effect of DMF on the distribution of non-follicular Th and follicular Tfh effector subpopulations between untreated and 6 and 12 months treated RRMS patients identified according to their expression of CXCR3 and CCR6 (Figure 5D).

When we compared non-follicular Th subpopulations, decrease in the percentage of Th1 cells in 12 months treated RRMS patients was observed once compared to the untreated group (15.3 vs. 25.3%; *p* < 0.01) (Figure 5E). Moreover, we found an increase in the percentages of Th2 cells in 6 months treated patients (49.8 vs. 35.8%; *p* < 0.01) and an even higher increase in 12 months treated patients (56.4 vs. 35.8%; *p* < 0.001) compared to the untreated group (Figure 5G). We also detected a decreased percentage of non-follicular Th17.1 cells in 6 months treated patients (8.5 vs. 14.9%; *p* < 0.05) and an even higher decrease in 12 months treated patients (6.3 vs. 14.9%; *p* < 0.001) compared to untreated RRMS patients (Figure 5H). No differences were found when the effect of DMF on effector Th17 cells subpopulation was evaluated (Figure 5F).

When the effect of DMF on follicular cTfh effector subsets was separately evaluated, a lower percentage of cTfh1 cells in 12 months treated patients compared to untreated patients was found (17.4 vs. 25.5%; *p* < 0.05) (Figure 5I). The percentage of cTfh2 cells progressively increased significantly upon DMF treatment, being at 6 months (51.6 vs. 37.3%; *p* < 0.01 and) and at 12 months (55.1 vs. 37.3%; *p* < 0.001) (Figure 5K). Since we had observed an increase in the CXCR3+CCR6+ follicular cTfh17.1 population in RRMS untreated patients compared to controls (Figure 2L), we tested whether DMF treatment could modulate this subpopulation. Interestingly, we found a decrease in the percentage of cTfh17.1 in 6 months treated patients compared to untreated patients (5.1 vs. 9.9%; *p* < 0.01) that was even higher after 12 months treatment (4.2 vs. 9.9%; *p* < 0.001) (Figure 5L). Again, no differences were found when cTfh17 subpopulations were compared (Figure 5J).

Remarkably, we identified three non-responders patients in our cohort in whom DMF treatment had to be discontinued (patients 6, 8, and 18). When lymphocyte subpopulations of these particular patients were separately revised, we observed that DMF treatment had modified non-follicular Th subsets, but had not normalized the basal deviation of cTfh subpopulations: patient 6 had 37% cTfh2 and 9% cTfh17.1, patient 8 had 41% cTfh2 and 9% cTfh17.1 (both evaluated at 6 months), and patient 18 had 32% cTfh2 and 11% cTh17.1 (evaluated at 12 months). In all cases, cTfh2 remained below percentile 25 (45%) and cTfh17.1 remained over percentile 75 (5%) of healthy subjects.

Moreover, patients 6 and 8 also maintained altered their B cells subpopulations upon 6 months DMF treatment. Patients 6 and 8 had 51 and 42% naïve B cells, respectively, both below percentile 25 of healthy subjects (52%). In addition, their switched memory B cells remained over percentile 75 of healthy subjects (19%), being 31 and 33%, respectively. Furthermore, these two patients (6 and 8) were the most affected at clinical evaluation on admission: EDSS > 3, more than 20 supratentorial lesions, ≥5 infratentorial lesions, and ≥2 spinal cord lesions in the MRI. Our data suggest that DMF may not be indicated for patients that are severely affected at disease onset.

### Longitudinal Study Confirms Cross-Sectional Data

Longitudinal data derived from 14 RRMS patients, who could be followed both before and after 6 and 12 months DMF treatment, were analyzed to validate our cross-sectional findings. Overall, both Th cells and cTfh cells percentages decreased with time upon DMF treatment, confirming cross-sectional results (data not shown).

**Figure 6:**
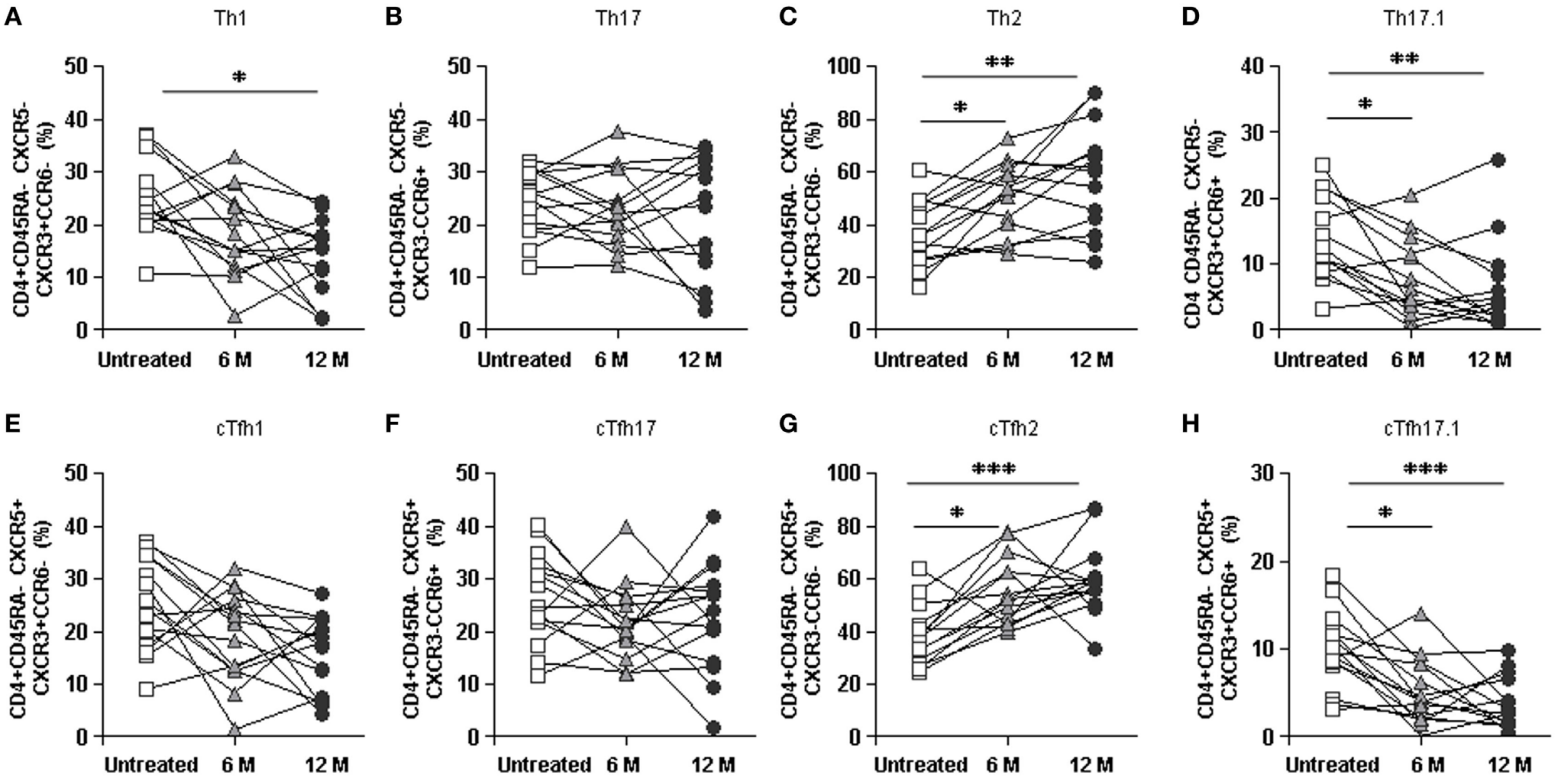
Longitudinal study of the effect of dimethyl fumarate on Th and cTfh subpopulations from multiple sclerosis (MS) patients. **(A–D)** Percentages of Th1, Th17, Th2, and Th17.1 non-follicular T cells and **(E–H)** cTfh1, cTfh17, cTfh2, and cTfh17.1 follicular T cells from MS patients, before treatment (white squares) and 6 months (6 M: light gray triangles) and 12 months (12 M: dark gray circles) after treatment. Successive repeated measures on the same individual are represented by connecting lines. Friedman paired test *p*-values: *p < 0.05, **p < 0.01, and ***p < 0.001.

Specifically, percentages of Th1, Th17.1, cTfh1, and cTfh17.1 cells subpopulations progressively decreased whereas Th2 and cTfh2 progressively increased along DMF treatment (Figures 6A–H). Percentages of Th17 and cTfh17 cells were not significantly altered during the longitudinal study (Figures 6B,F), confirming the data obtained in the cross-sectional study (Figures 5E–L).

### DMF Reverts the Pro-Inflammatory Shift of cTfh and B Cells to Healthy Subject’s Values

To evaluate the final effect of DMF treatment, we compared the percentages of non-follicular Th, follicular cTfh effector, and B cells subpopulations on the RRMS group of patients at the end of the 12 month treatment period with those of normal healthy subjects.

**Figure 7:**
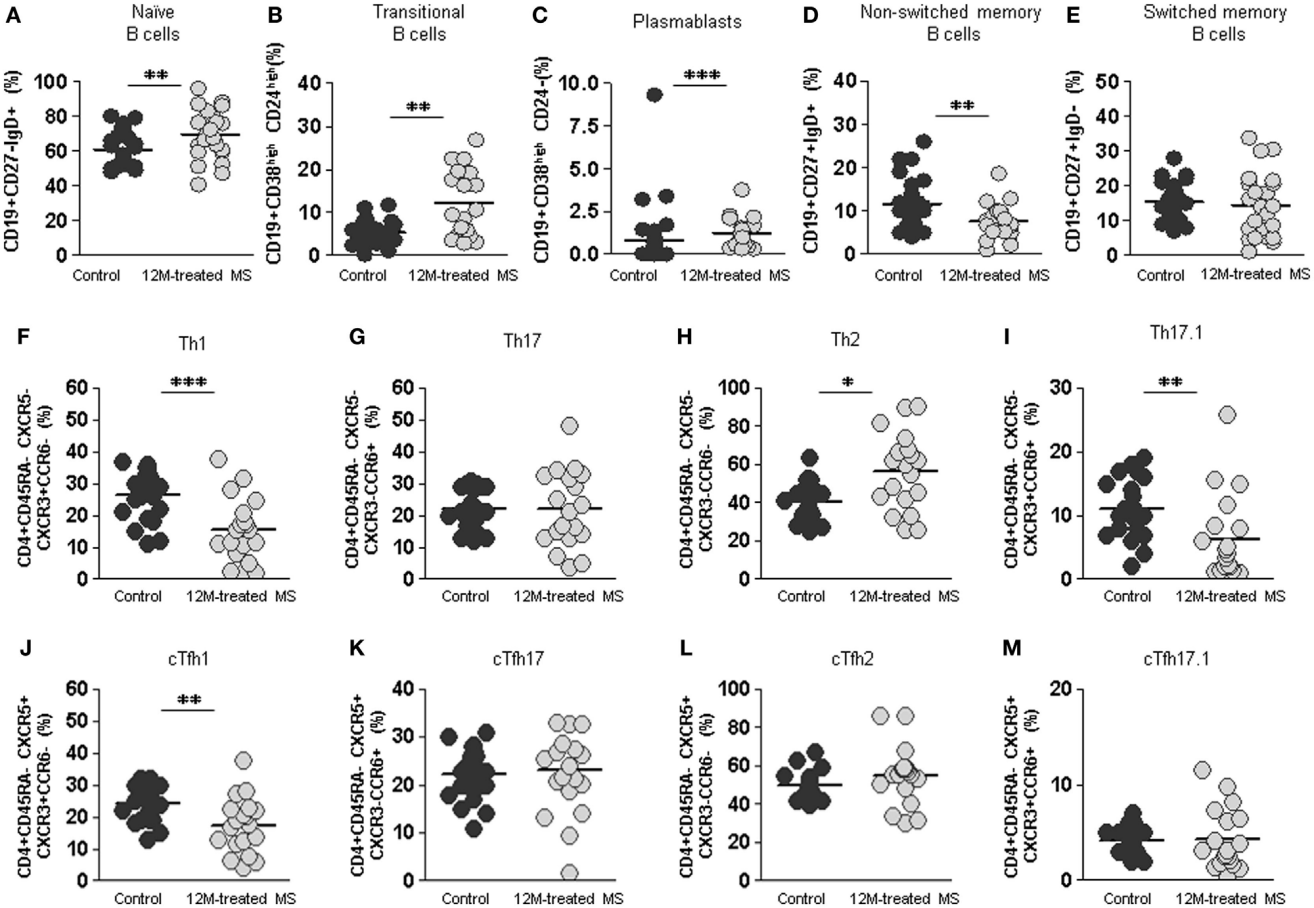
Final effect of dimethyl fumarate on B cell, non-follicular Th cells, and follicular cTfh cells subpopulations compared to healthy controls. **(A–E)** Percentages of naïve CD19+CD27−IgD+, transitional CD19+CD38^high^CD24^high^, plasmablasts CD19+CD38^high^CD24−, non-switched memory CD19+CD27+IgD+, and switched memory CD19+CD27+IgD− B cells from healthy controls (dark gray circles) and 12 months treated multiple sclerosis (MS) patients (12M: light gray circles). **(F–I)** Percentages of Th1, Th17, Th2, and Th17.1 non-follicular T cells and **(J–M)** percentages of cTfh1, cTfh17, cTfh2, and cTfh17.1 follicular T cells from healthy controls (dark gray circles) and 12 months treated MS patients (12M: light gray circles). Mann–Whitney *U*-test *p*-values: *p < 0.05, **p < 0.01, and ***p < 0.001.

Remarkably, at this point of treatment, initially altered percentages of switched memory (CD19+CD27+IgD−) B cells returned to values of healthy subjects (Figure 7E). This was accompanied by a decrease of non-switched memory (CD19+CD27+IgD+) B cells to values significantly lower than those of healthy subjects (7.6 vs. 11.6%; *p* < 0. 01) (Figure 7D). On the contrary, we found a significant increase in naïve (69.5 vs. 60.7%; *p* < 0.01), transitional (12.3 vs. 5.3%; *p* < 0.01), and plasmablasts (0.8 vs. 1.2%; *p* < 0.001) B cells at the end of the studied treatment period (Figures 7A–C).

After 12 month DMF treatment, percentages of both Th1 and Th17.1 non-follicular cells in 12 month treated RRMS group were even lower than those of healthy controls (15.3 vs. 26.4%; *p* < 0.001 and 6.3 vs. 11.1%; *p* < 0.01, respectively). Conversely, percentages of Th2 subpopulation were increased in the 12 month treated RRMS group compared to controls (56.4 vs. 40.3%; *p* < 0.05). This is consistent with an anti-inflammatory switch in non-follicular Th subpopulations induced by DMF treatment (Figures 7F–I).

When we evaluated effector follicular cTfh cells, cTfh1 were lower in the 12 month treated RRMS group compared to healthy controls (17.4 vs. 24.1%; *p* < 0.01); meanwhile, the percentages of cTfh2, cTfh17.1 did not differ between 12 month treated RRMS group and healthy controls (Figures 7J–M).

Thus, DMF reduces the absolute numbers of all major lymphocyte subpopulations, reverts the pro-inflammatory shift of the relevant cTfh and switched-memory B cells detected in untreated RRMS patients, and exerts a modifying effect in naïve, transitional, plasmablasts, and non-switched memory B cells subpopulations percentages.

## Discussion

Several immunological components have been implicated in the pathogenesis of MS with special relevance for CD4+ T cells (1), although an important role for B lymphocytes has also been demonstrated (6). We investigated the frequency and distribution of different lymphocyte subpopulations, with special focus on cTfh cells, in RRMS patients compared to healthy subjects. Moreover, we evaluated whether these subpopulations could be modified in response to DMF treatment, and whether this potential shift could associate to treatment response in RRMS patients.

Although percentages and absolute counts of peripheral CD4+ and CD8+ T, NK, and B cells in our cohort of untreated RRMS patients were within reported ranges, distribution of B cells subsets was altered: the percentage of switched-memory B cells was increased.

cTfh cells have been previously found increased in MS patients (23) and ectopic lymphoid structures containing Tfh cells and B cells have been described in the meninges of MS patients, which could contribute to disease pathogenesis (5). Although we studied the subpopulations of non-follicular and Tfh cells, in our cohort of untreated RRMS patients, we only found important differences in the distribution of cTfh cells subpopulations. RRMS patients presented higher percentage of cTfh17.1 cells and lower percentage of cTfh2 cells, consistent with a pro-inflammatory bias only in cTfh subpopulations. cTfh17.1 cells express both CXCR3+CCR6+ and are analogous to the recently described Th17.1 helper effector subpopulation that produces high levels of IFNγ and IL-17 (16). Remarkably, Th17.1 subpopulation is resistant to glucocorticoids (16) and is increased in Crohn’s disease (17) and in the lungs of sarcoidosis patients (16, 18).

Controversy exists about the implication of Th subpopulations and the role of IL-17 and IFNγ in the pathogenesis of MS. In RRMS patients, IL-17 levels were higher in serum and CSF (24) and IL-17-expressing CD4+ T cells were increased during relapses, while IFNγ-expressing CD4+ T cells remained stable (25). Moreover, myelin oligodendrocyte glycoprotein-specific CD4+ T cells in blood of RRMS patients were mostly CCR6 memory cells (5) producing higher levels of IFNγ, IL-17, and GM-CSF (26). Experiments in the experimental autoimmune encephalomyelitis MS mouse model have shown that Th17 cells induce ectopic lymphoid structures in the subarachnoid space, where they acquire a Tfh phenotype (27) and also that Th cells producing both IFNγ and IL-17 were more pathogenic than Th cells producing IFNγ or IL-17 alone (5). The fact that we found an increase in cTfh17.1 but not in cTfh17 may help to explain these controversies, as cTfh17.1 cells are able to produce both IFNγ and IL-17 cytokines. We suggest that the increase in cTfh17.1 and decrease in cTfh2 cells observed in our patients cohort may have a role in the pathogenesis of RRMS disease and could be a potential treatment target. Previous studies on other autoimmune diseases have also suggested that a skewed distribution of cTfh cell subsets contributes to their pathogenesis: higher levels of Tfh17 in primary Sjögren’s syndrome (28) and also increased levels of both Tfh2 and Tfh17 in juvenile dermatomyositis (12) and Guillain–Barré syndrome (29).

Dimethyl fumarate is an approved oral treatment for RRMS. Recently, several articles addressing the effect of DMF in peripheral lymphocytes subpopulations have been published with the aim to underscore its mechanism of action and/or to find biological markers predicting treatment response. A reduction in lymphocyte counts and selective reductions in CD8+ T cells (30) or memory T cells (31) have been described after DMF treatment. An anti-inflammatory shift in B cells subsets has also been described; with decrease of the memory B cells pool and reduction in GM-CSF, IL-6, and TNFα expressing B cells (32).

Our 29 patients cross-sectional study confirmed a decrease in the absolute numbers but not in the percentages of CD4+ and CD8+ T cells, B, and NK cells, consistent with the lymphopenia described in DMF-treated patients (9). However, an important shift was detected in B cells subpopulations distribution: while switched memory B cells significantly decreased, a significant increase of naïve, transitional, and plasmablasts B cells was observed, whereas non-switched memory B cells remained unaltered.

We observed that DMF induced a progressive decrease of non-follicular Th1 and an increase in the anti-inflammatory Th2 subpopulation, whereas Th17 subpopulation remained unchanged, suggesting an anti-inflammatory shift in previously unaltered Th subpopulations. Our results are consistent with previous reports in which a decrease in Th1, increase in Th2, and no alteration of Th17 memory T cells was reported in 15 RRMS patients treated only 6 months with DMF (33) A reduction in Th1 (analyzed as CXCR3+) and increase in Th2 (analyzed as CCR3+) frequencies were also reported in a nine patients longitudinal cohort (9). However, in contrast to our study, these authors also found a reduction in Th17 cells. Discrepancy in the results may be due to the experimental protocol used to analyze Th17 subpopulation: their analysis was based on “CCR6+ only,” which presumably includes CCR6+CXCR3− non altered Th17 cells, but also our separately analyzed CCR6+CXCR3+ Th17.1 cells, the subpopulation which we have identified to be reduced by DMF treatment.

Our most relevant finding comes from the study of cTfh subpopulations, which are responsible for driving B cell differentiation. Important differences were found between untreated and DMF-treated patients: a significant increase of cTfh2 cells was observed in 12 month treated patients, accompanied by a significant decrease in cTfh1 and cTfh17.1 cells. No differences were found in cTfh17 percentages. These results imply that DMF reverts the pro-inflammatory switch of cTfh cells detected in untreated RRMS patients. B cells modifications found after treatment could be an indirect consequence of the observed cTfh subpopulations modulation, but we cannot rule out a direct effect of DMF on B cells.

In any case, DMF-induced changes in these subpopulations had a humoral consequence. Although serum levels of IgG and IgM were not importantly modified, IgA significantly decreased over time in response to DMF treatment. This is an important observation as a recent report has demonstrated a relationship between CSF IgA levels and IgA CSF/serum quotient, and cerebral atrophy and EDS in MS patients (34). Moreover, given the importance of several cytokines, particularly IFNγ, in the IgG subclass switch process (35, 36), changes in IgG subclass profile (decrease in IgG3 and IgG2) in the serum of our DMF-treated patients favor a role of the Tfh subpopulations shift and their secreted cytokines in the DMF treatment effect.

When we compared untreated RRMS patients with healthy subjects, we only found differences in the cTfh subpopulations (higher percentage of Tfh17.1 and lower percentage of Tfh2 cells) and B cells subpopulations (increase in switched memory B). Over the course of our study, DMF progressively reverted this proinflammatory shift in cTfh and B cells subpopulations by the end of the 12-month treatment if compared to healthy controls values. Interestingly, three DMF non-responder patients did not revert the deviation of cTfh subpopulations. Although these findings should be validated with wider cohorts of DMF non-responders, our data suggest that normalization of cTfh subpopulations upon DMF treatment may be related to clinical response. Moreover, the fact that two of these patients were the most severely affected at baseline evaluation may suggest that DMF is not effective in those cases and treatment should be started earlier.

In summary, we have demonstrated a pro-inflammatory shift of cTfh and B cells in RRMS patients. DMF treatment induces a progressive decrease in cTfh1 and the pathogenic cTfh17.1 subpopulations, together with an increase in cTfh2 subpopulations, resulting in a reversion of this situation. This effect is accompanied by a corresponding decrease in switched-memory B cells and an increase in naïve, transitional, and plasmablasts B cells. We postulate that the decrease in pro-inflammatory IFNγ and IL-17 producing cells and antigen experienced B cells may explain the protective activity of DMF in RRMS-treated patients. The measurement of cTfh subpopulations could be a biological marker to evaluate DMF response.

## Ethics Statement

The study was conducted according to the ethical guidelines of the 1975 Declaration of Helsinki and approved by CEIC (Balearic Islands Clinical Research Ethics Committee). Written informed consent was obtained from all subjects.

## Author Contributions

VC performed the experiments and conducted the acquisition, interpretation, and statistical analysis of data. MM, CC, VN, and RD conducted patients recruitment and clinical information monitoring. AC collaborated with the analysis and interpretation of data and critical revision of the manuscript. VA, AL-G, and MJ collaborated with the acquisition and analysis of data. JP collaborated with the critical revision of the manuscript. CV-B designed research project and collaborated with the critical revision of the manuscript. JF designed research project, generated funding, and wrote the manuscript.

## Conflict of Interest Statement

VC, AC, VA, AL-G, RD, MJ, JP, CV-B, and JF declare no conflict of interest. MM, CC, and VN have been speakers on behalf of Biogen.
